# Supplementary material for: Climate change will reduce North American inland wetland areas and disrupt their seasonal regimes
Source: Nat Commun. 2024 Mar 18;15:2438. doi: 10.1038/s41467-024-45286-z (PMC10948824; doi:10.1038/s41467-024-45286-z)
Supplement: Supplementary file 1 — Supplementary Information [file 41467_2024_45286_MOESM1_ESM.pdf]

Supplementary Information for

Climate change will reduce North American inland wetland areas and disrupt their seasonal regimes

Donghui Xu[1,*], Gautam Bisht[1,*], Zeli Tan[1], Eva Sinha[1], Alan V. Di Vittorio[2], Tian Zhou[1], Valeriy Y. Ivanov[3], L. Ruby Leung[1]

[1]Atmospheric Sciences and Global Change Division, Pacific Northwest National Laboratory, Richland, WA, USA
[2]Earth and Environmental Sciences Area, Lawrence Berkeley National Laboratory, Berkeley, CA, USA
[3]Department of Civil and Environmental Engineering, University of Michigan, Ann Arbor, MI, USA

Correspondence to: Donghui Xu, donghui.xu@pnnl.gov,

Gautam Bisht, gautam.bisht@pnnl.gov

This file includes:

Supplementary Text 1
Supplementary Figure 1 to Supplementary Figure 19
Supplementary References

Supplementary Text 1. Pluvial inundation scheme in ELM

Surface water storage component was introduced in land surface models to aid the simulation of the pluvial inundation process [1, 2]. However, ad-hoc calibration is necessary to constrain the parametric uncertainty since sub-grid parameterizations are needed to compensate for the typically coarse resolution of ESMs. Furthermore, satellite observations used to validate wetland dynamics cannot differentiate between fluvial and pluvial inundation, calling for process-based model simulations that can adequately mimic the two inundation types.

The functions of surface water storage and outflow are determined by microtopography:

$$W_{sfc} = \frac{d}{2}\left(1 + \mathrm{erf}\left(\frac{d}{\sigma_{micro}\sqrt{2}}\right)\right) + \frac{\sigma_{micro}}{\sqrt{2\pi}}\, e^{\frac{-d^2}{2\sigma_{micro}^2}}, \tag{1}$$

where W_{sfc} is the mass of the surface water $[kg \cdot m^{-2}]$, erf represents the error function, d is the height of surface water relative to the cell averaged elevation $[m]$, and σ_{micro} is the standard deviation of the microtopographic distribution, $[m]$, characterizing the sub-grid elevation variation. Since microtopography information is not available at a large scale, the parameterization of σ_{micro} is proposed in Supplementary ref. [1]:

$$\sigma_{micro} = \left(\beta + (\sigma_{max})^{\frac{1}{\eta}}\right)^{\eta}, \tag{2}$$

where β is the topographic slope, σ_{max} represents the maximum value of σ_{micro}, and η is an adjustable parameter. The uncertainty of σ_{max} and η is not the focus of this study, so we used literature default values of $\sigma_{max} = 0.4$ and $\eta = -3$ [1]. Given the surface water height from the previous equation, one can estimate the inundation fraction (f_{h2osfc}) of the cell with:

$$f_{h2osfc} = \frac{1}{2}\left(1 + \mathrm{erf}\left(\frac{d}{\sigma_{micro}\sqrt{2}}\right)\right), \tag{3}$$

The surface water storage functions as a linear reservoir when the f_{h2osfc} is larger than a threshold (f_c) and the outflow ($q_{out,h2osfc}$) is estimated as:

$$q_{out,h2osfc} = k_{h2osfc} f_{connected} (W_{sfc} - W_c) \frac{1}{\Delta t}, \tag{4}$$

where k_{h2osfc} represents the linear storage coefficient, W_c is the surface water mass corresponding the threshold fraction, Δt is the time step, and $f_{connected}$ is the fraction of connected inundated areas based on percolation theory:

$$f_{connected} = \left(f_{h2osfc} - f_c\right)^{\mu}, \tag{5}$$

where μ is an exponent parameter. The values of f_c and μ are set to be 0.4 and 0.14 in default configuration, respectively.

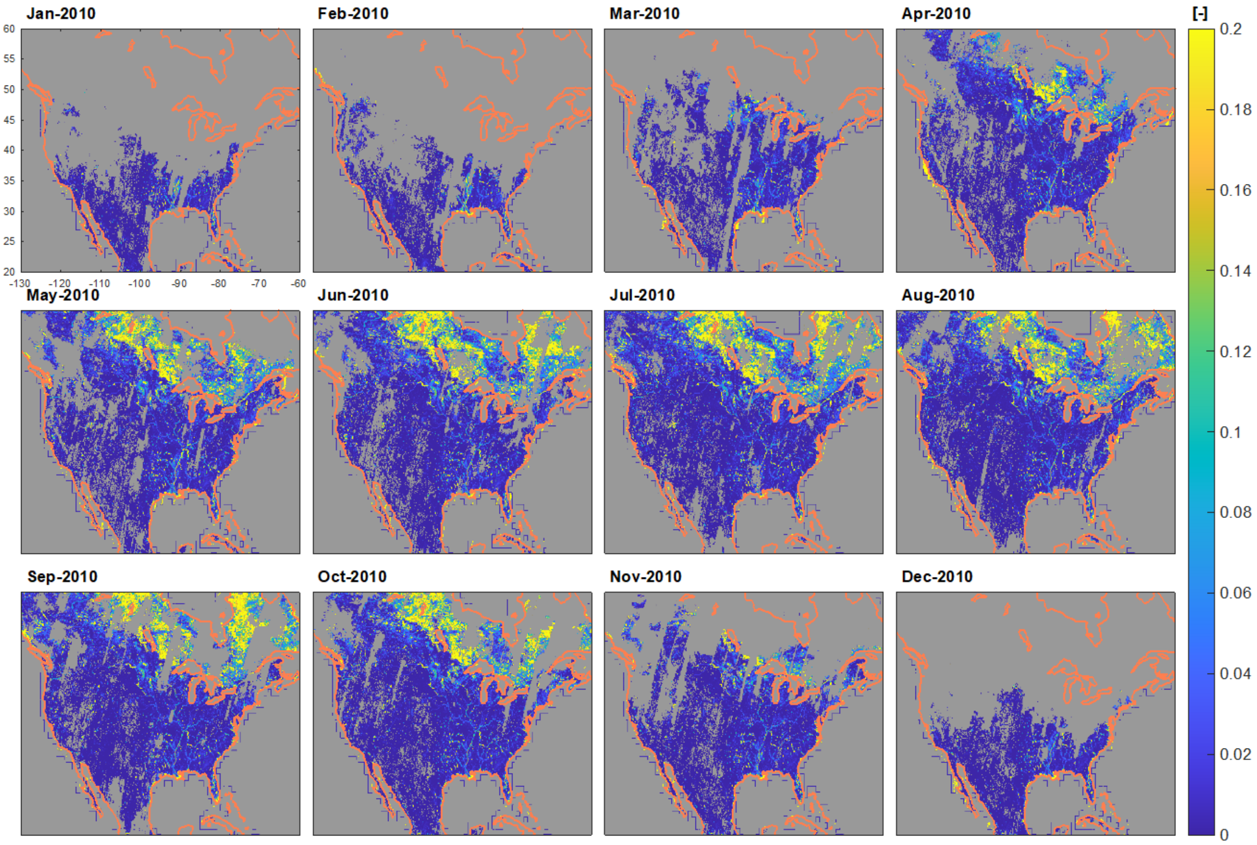

Supplementary Figure 1: Monthly upscaled GLAD surface water fraction [-] of 2010. The grey area represents grid cell that does not have a value.

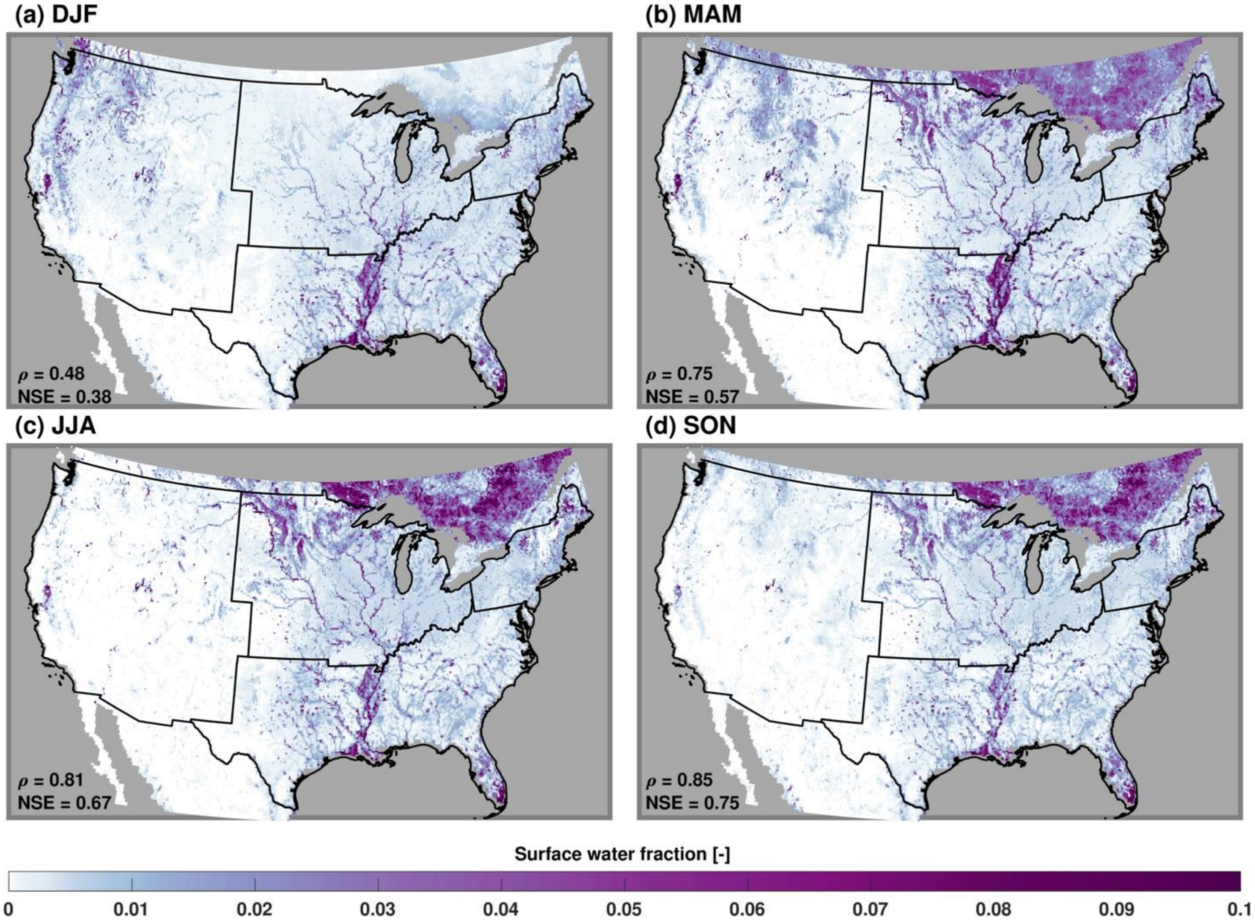

Supplementary Figure 2: Evaluation of the simulated surface water fraction with coupled ELM-MOSART configuration over 1999-2020 period for (a) winter, (b) spring, (c) summer, and (d) fall. The inset text in each subplot shows evaluation metrics by comparing the simulation results with the upscaled Global Land Analysis & Discovery (GLAD) surface water fraction for each season with permanent surface water removed. Symbols ρ and NSE are the correlation coefficient and the Nash–Sutcliffe model efficiency coefficient, respectively. DJF represents December, January, and February. MAM represents March, April, and May. JJA represents June, July, and August. SON represents September, October, and November. The grey color denotes no available data.

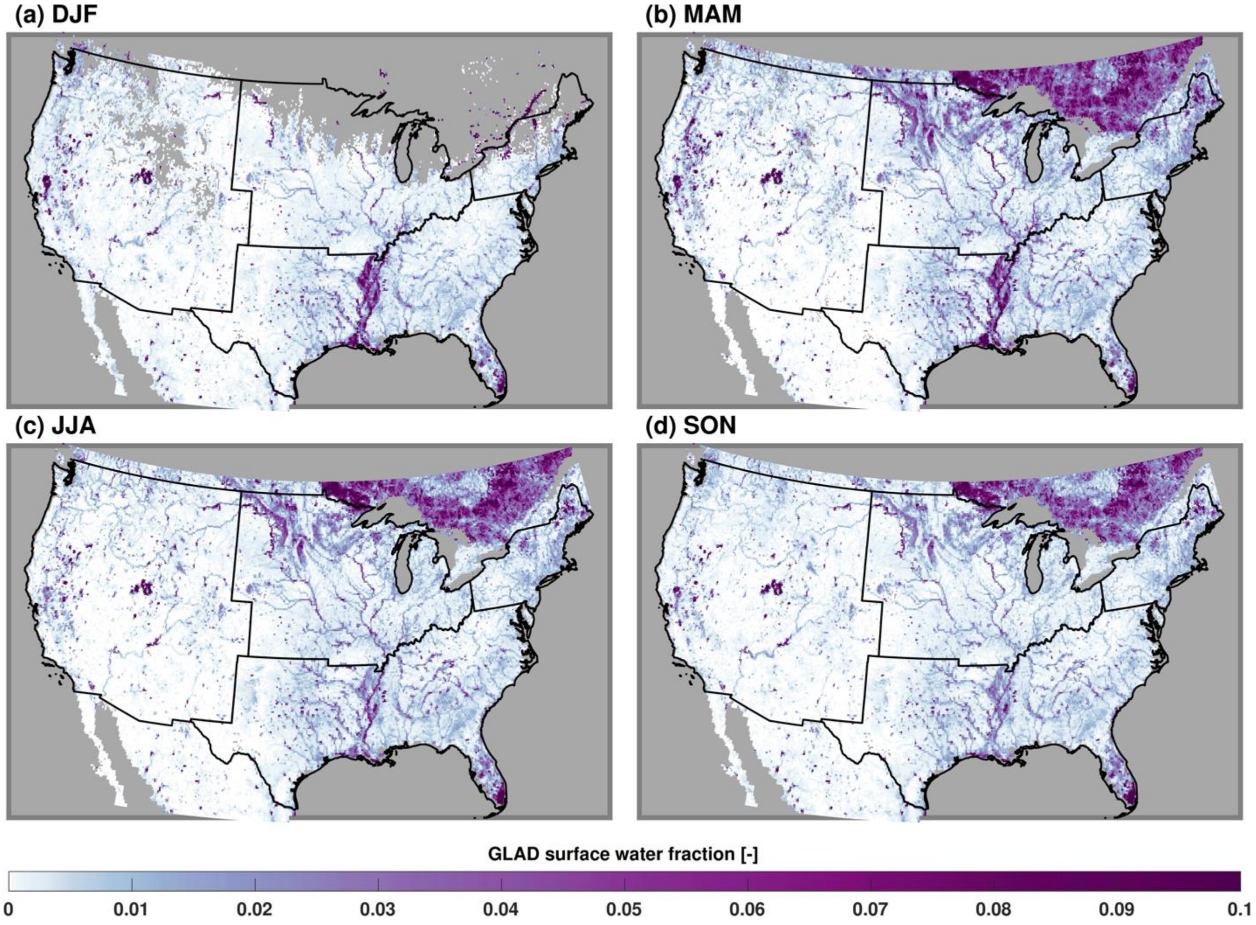

Supplementary Figure 3: Upscaled GLAD seasonal surface water fraction during 1999-2020 with permanent water fraction excluded for (a) winter, (b) spring, (c) summer, and (d) fall. DJF represents December, January, and February. MAM represents March, April, and May. JJA represents June, July, and August. SON represents September, October, and November. The grey area represents no available data.

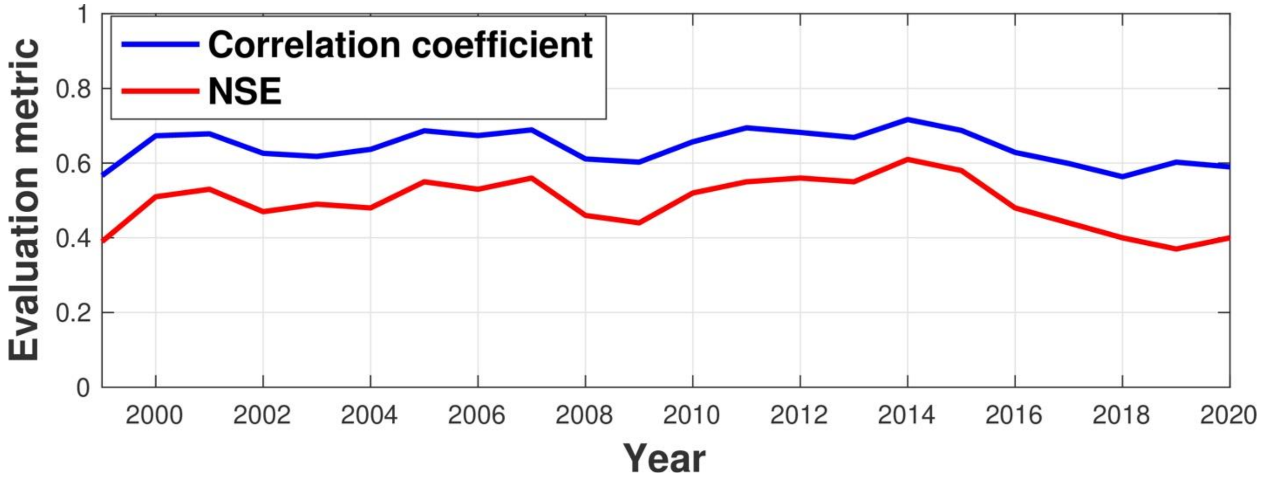

Supplementary Figure 4: Evaluation metrics comparing E3SM simulated surface water and GLAD surface water with permanent water bodies removed at the annual time scale. The evaluation metric is estimated based on the comparison between simulation and observation for all the grid cells in the study domain.

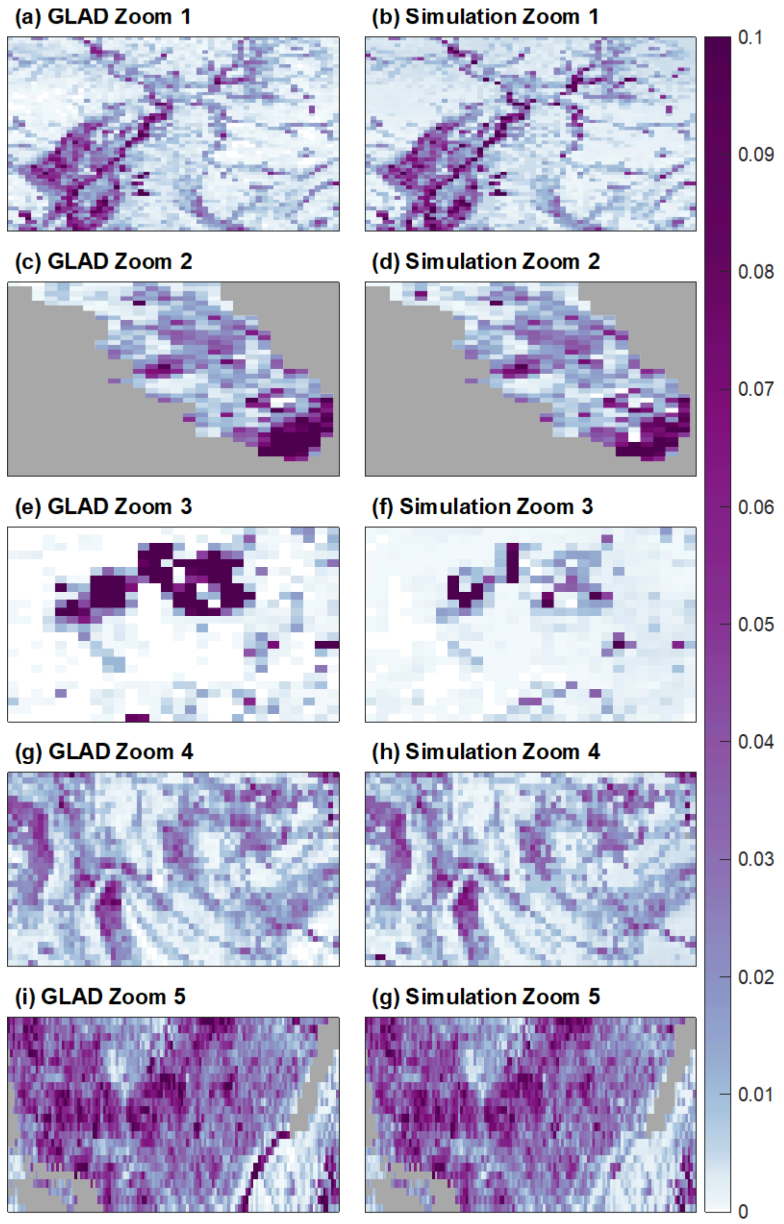

Supplementary Figure 5: Comparison of simulated surface water (unit: fraction of a grid cell) with upscaled GLAD surface water observation (with permanent surface water removed) averaged over 1999-2020 for the zoomed-in regions. (a), (c), (e), (g), and (i) show the GLAD dataset for five zoomed-in regions delineated in Supplementary Figure 19. (b), (d), (f), (h), and (g) are the corresponding simulations.

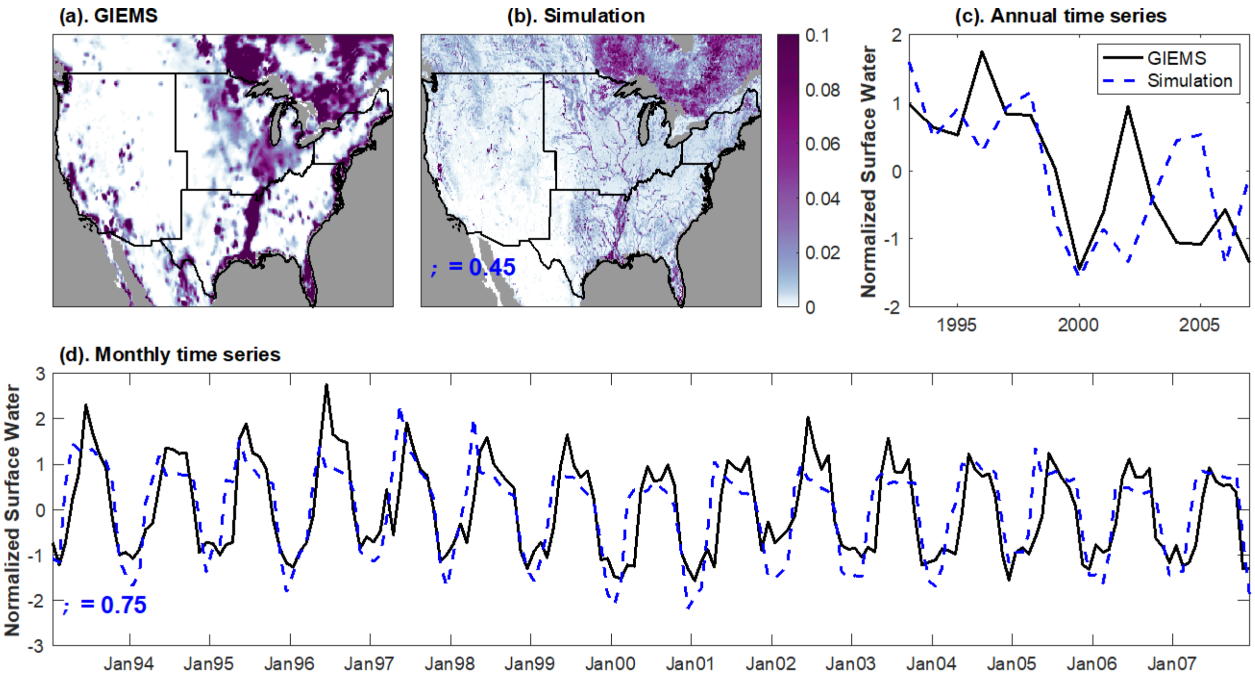

Supplementary Figure 6: **Evaluation of simulated surface water (unit: fraction of a grid cell) with satellite observation from Global Land Analysis & Discovery (GLAD) averaged over 1993-2007.** (a) Averaged GIEMS inundated fraction from 1993 to 2007; (b) Simulated MOSART and ELM inundated fraction from 1993 to 2007; (c) and (d) illustrates the continent-averaged normalized wetland area (subtracting the mean and divided by the standard deviation) comparison between the simulation and GIEMS at annual and monthly scales, respectively. ρ in subplot (b) and (d) represent correlation coefficient. The grey color denotes no available data. We note GIEMS includes all the permanent surface water types, including lakes, rivers, reservoirs, ponding waters, and other permanent wetlands. However, GIESM doesn't provide permanent surface water. Therefore, we normalized both GIEMS and simulations to focus on the validation of temporal variability at monthly scales and interannual variability.

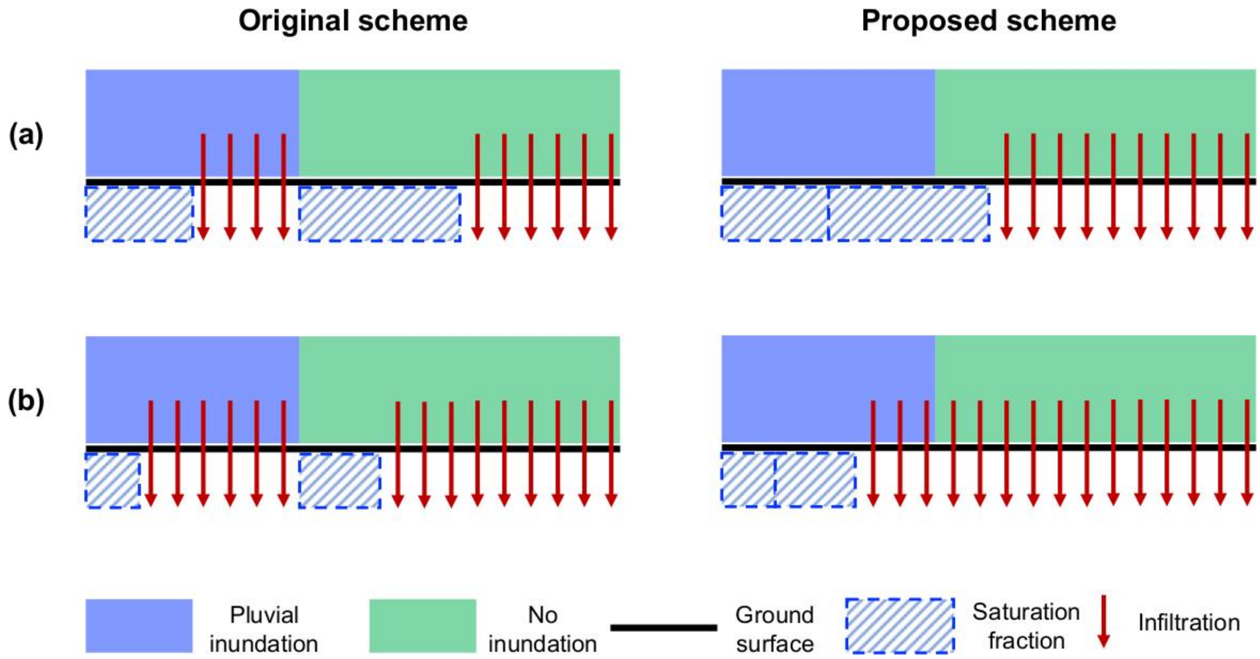

Supplementary Figure 7: The original infiltration scheme (left column) and the proposed infiltration scheme (right column) in E3SM land model. Subplot (a) represents the situation where pluvial inundation is less than the estimated saturation fraction and subplot (b) represents pluvial inundation larger than the saturation fraction. In the original formulation, saturated areas and infiltration process are considered to be homogeneously distributed, in proportion to a given land type, including inundated and non-inundated areas. In the new formulation, the saturation fraction is assigned only to the areas with inundation and infiltration occurs in the rest of the subgrid area.

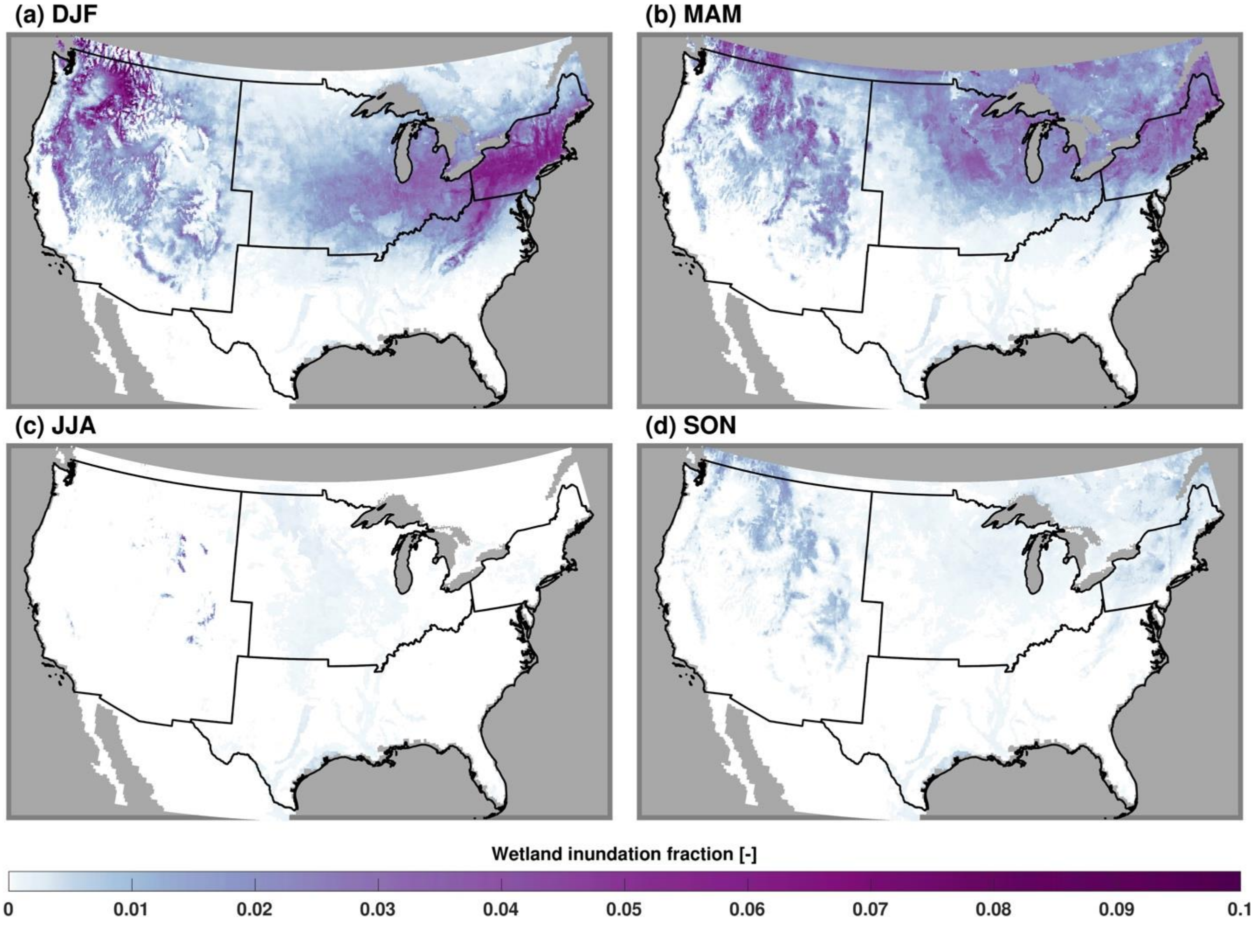

Supplementary Figure 8: ELM-simulated inundation with default inundation scheme during 1999-2020 for (a) winter, (b) spring, (c) summer, and (d) fall. DJF represents December, January, and February. MAM represents March, April, and May. JJA represents June, July, and August. SON represents September, October, and November. The grey color denotes no available data.

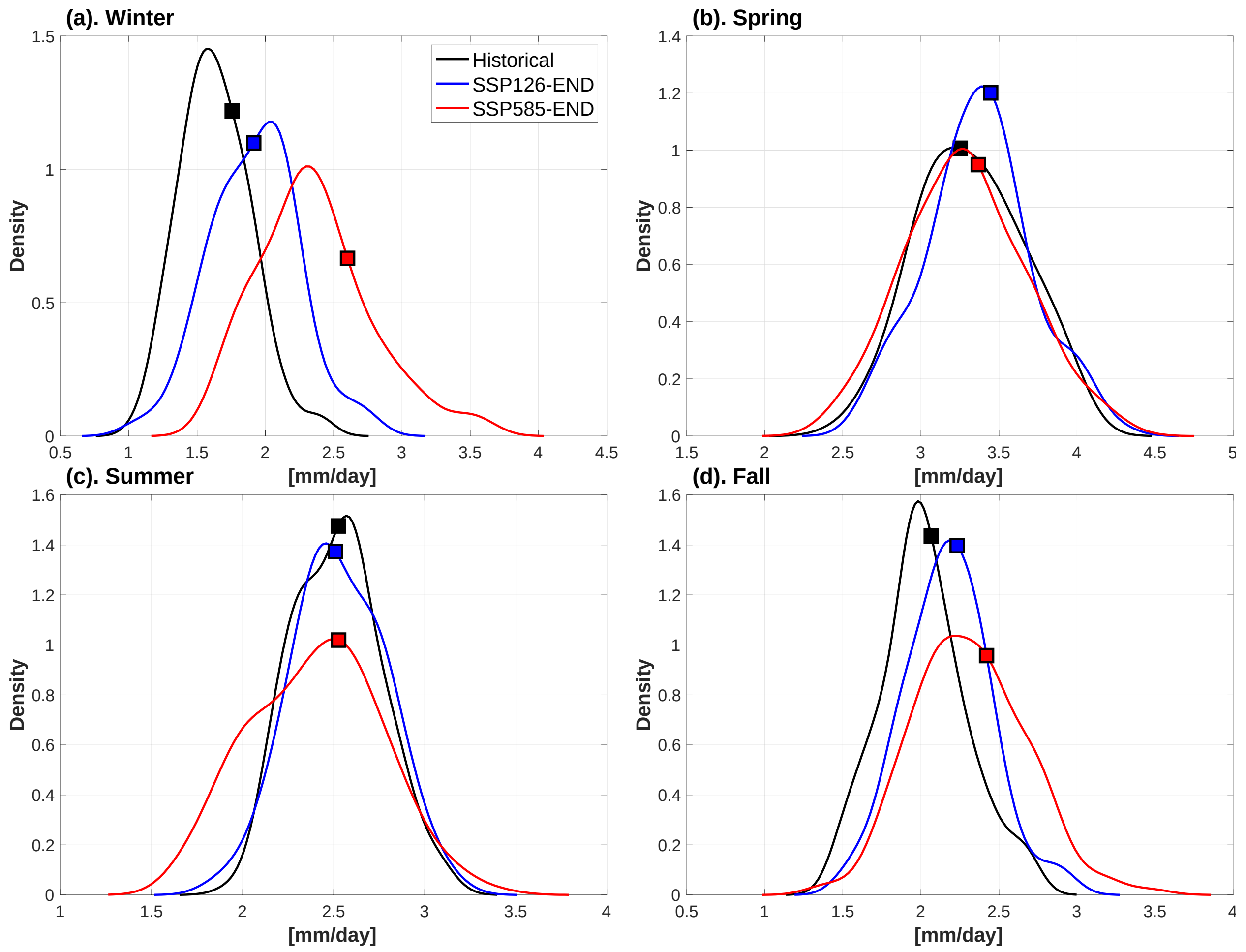

Supplementary Figure 9: **Probability distribution function (PDF) of liquid precipitation + snowmelts during (a) winter, (b) spring, (c) summer, and (d) fall.** The square scatters in each subplot represent the corresponding median of the PDF.

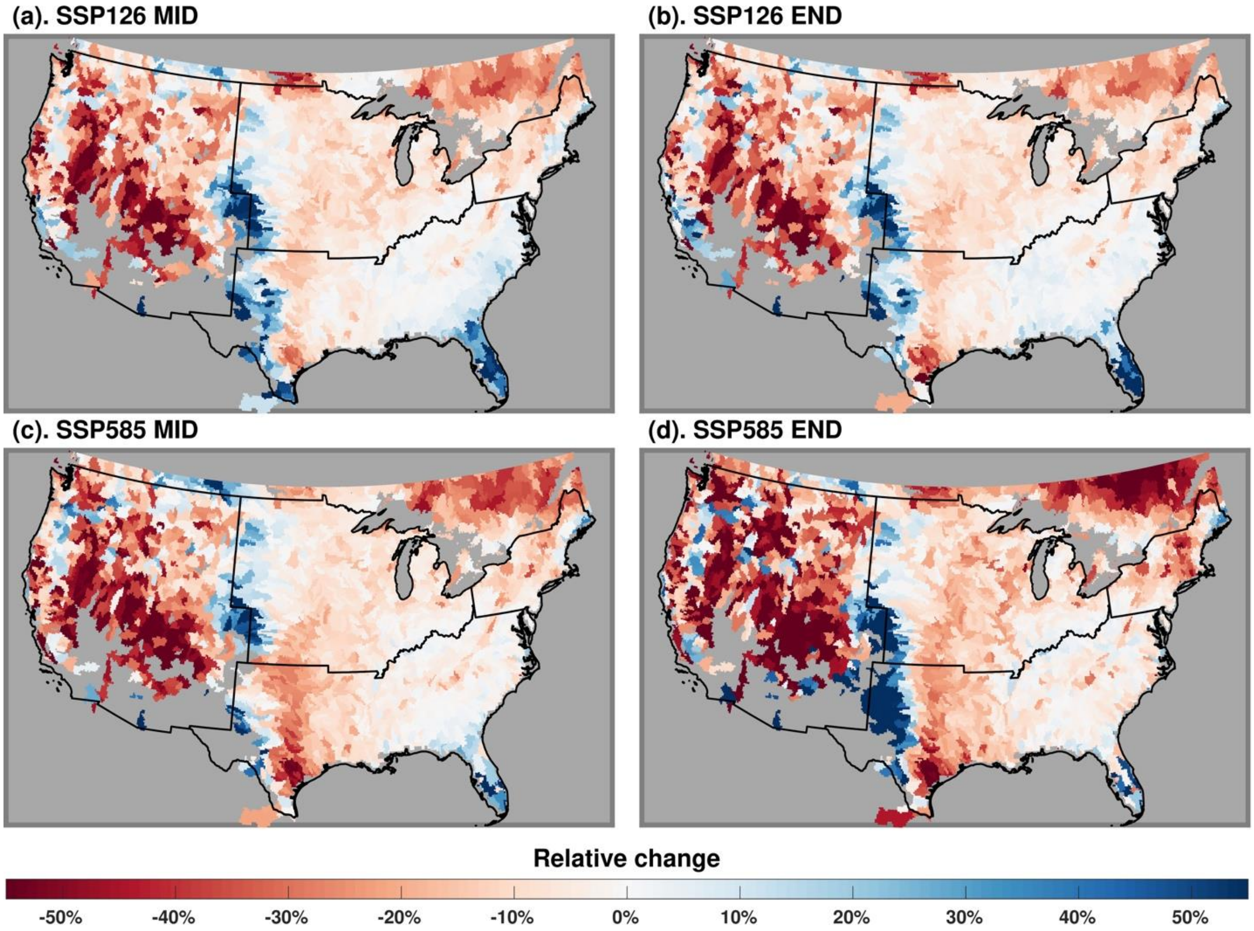

Supplementary Figure 10: Relative change of the mean annual wetland fraction between the historical (HIS) and future (FUT) periods with winter months (Dec, Jan, and Feb) excluded. The relative difference ($\frac{FUT - HIS}{HIS} \times 100\%$) is estimated as the mean of the equal-weighted multi-model ensemble. "MID" (subplots (a) and (b)) represents the relative change between the 2041-2070 and 1971-2000 periods. "END" (subplots (c) and (d)) represents the difference between 2071-2100 and 1971-2000 periods. The grey color denotes either no available data or the area that has negligible surface water in the historical period (i.e., less than 0.05%). All of the results are shown at the basin scale (See Materials and methods).

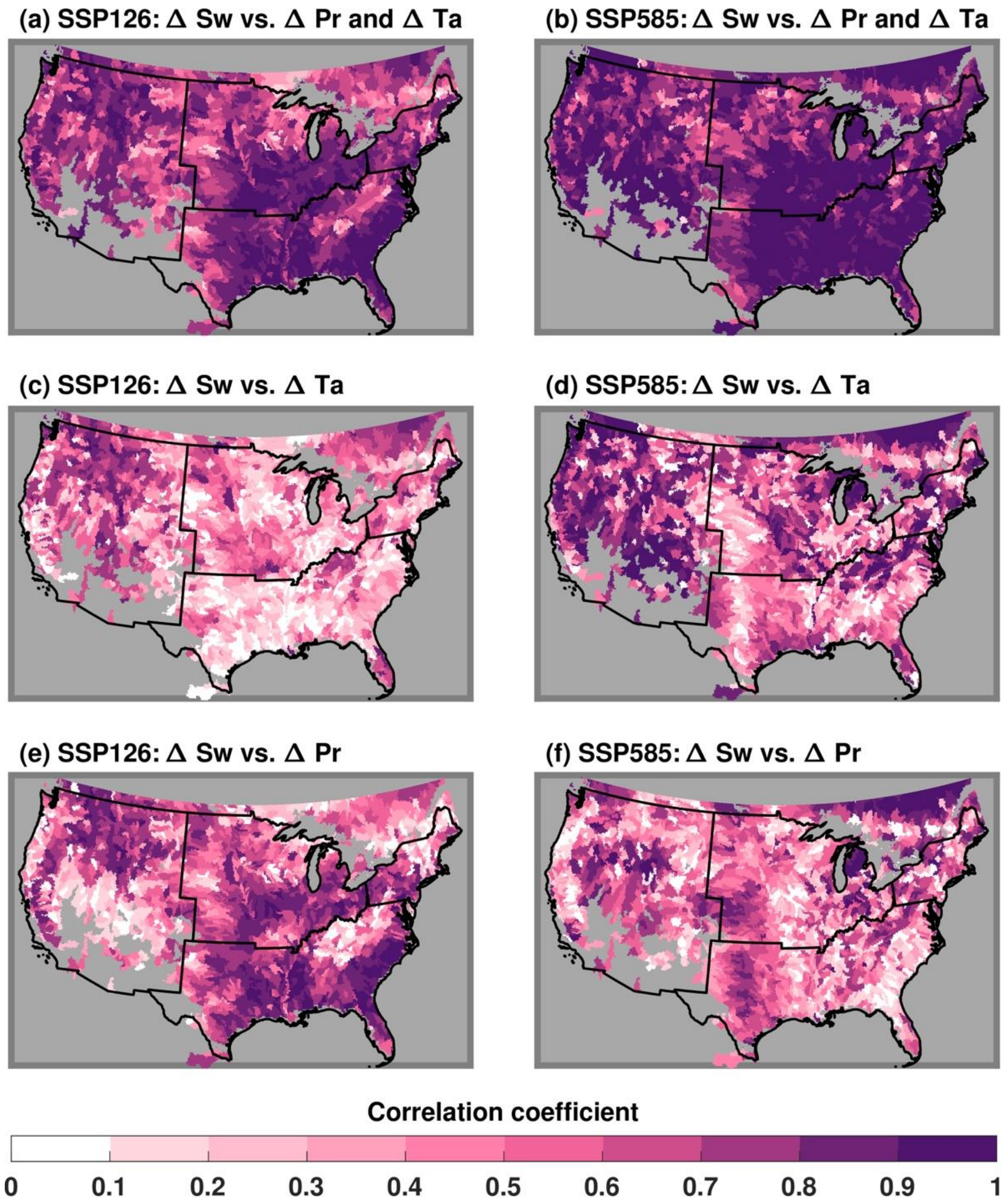

Supplementary Figure 11: Multilinear regression analysis for the change of surface water fraction. Correlation coefficients for the (a) multilinear regression between the surface water fraction change (ΔSw), precipitation change (ΔPr), and temperature change (ΔTa) in SSP126; (b) multilinear regression between ΔSw, ΔPr, and ΔTa in SSP585; (c) linear regression between ΔSw and ΔTa in SSP126; (d) linear regression between ΔSw and ΔTa in SSP585; (e) linear regression between ΔSw and ΔPr in SSP126; and (f) linear regression between ΔSw and ΔPr in SSP585. ΔSw, ΔPr, and ΔTa are multi-model ensemble means of the differences between the future (2015-2100) and historical period (1971-2000). Specifically, the historical mean of each quantity is removed from the future annual time series and smoothed with a 10-year moving average. The multi-model ensemble mean is the arithmetic mean of the five selected climate models. The grey color denotes either no available data or the area that has negligible surface water in the historical period (i.e., less than 0.05%)

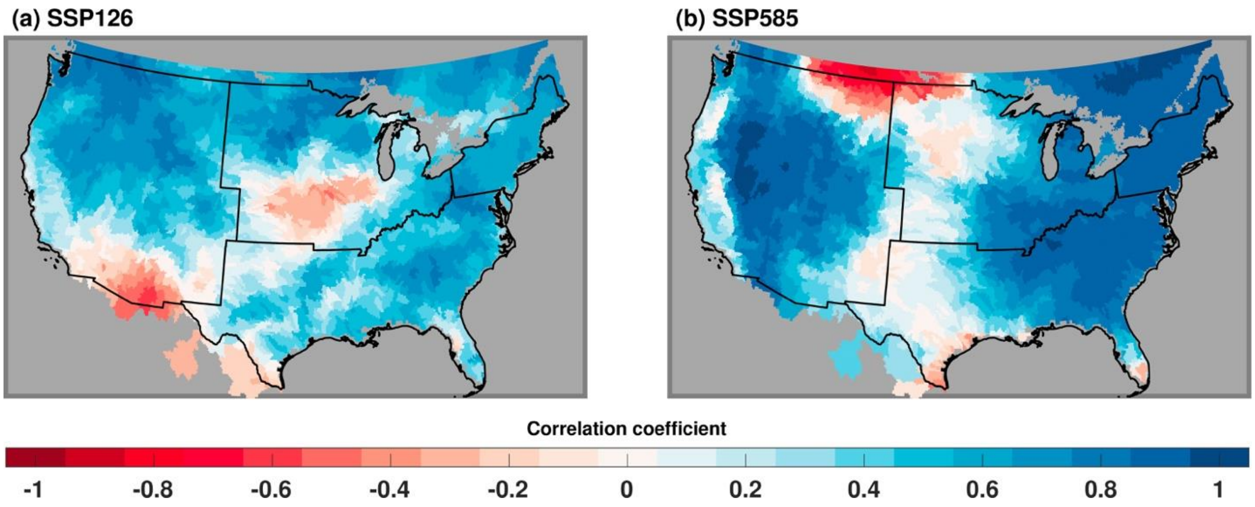

Supplementary Figure 12: Correlation coefficients between the change of temperature and change of precipitation for the entire future period (i.e., 2015-2100) in (a) SSP126 and (b) SSP585. The correlation coefficients were estimated with a multi-model ensemble of temperature changes and precipitation changes for each basin. Annual changes of temperature and precipitation were derived by removing the historical mean of 1971-2000 from the future annual time series and smoothed with a 10-year moving average. The multi-model ensemble mean is the arithmetic mean of the five selected climate models.

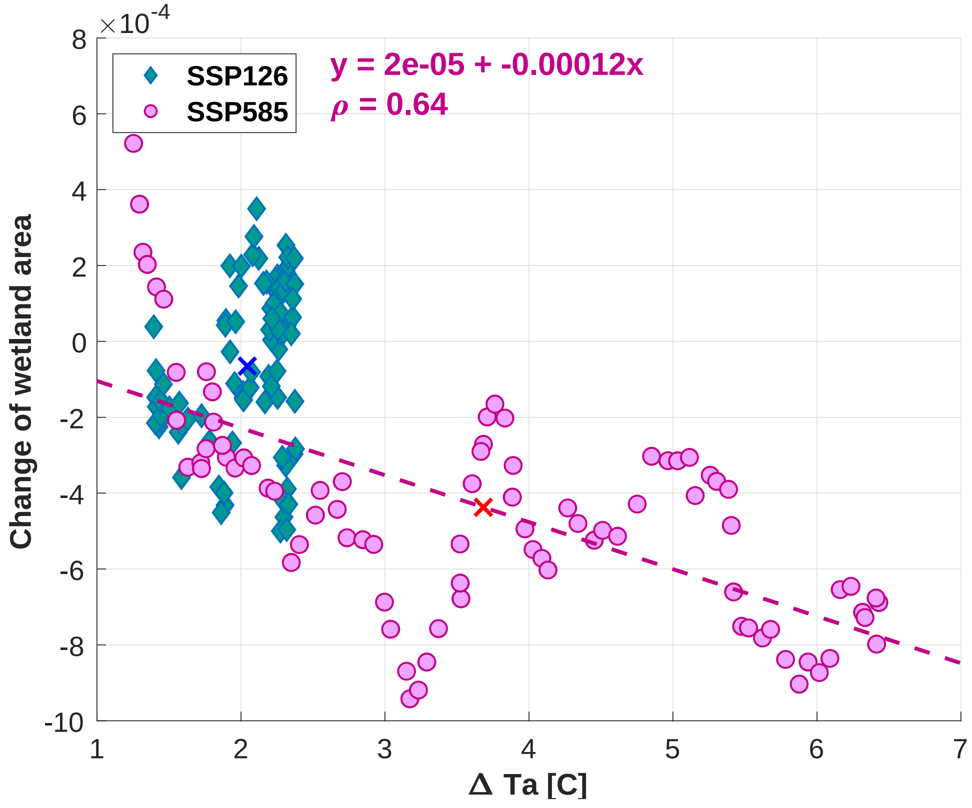

Supplementary Figure 13: Scatter plots between change of temperature (ΔTa, X-Axis) and change of wetland area (Y-Axis) for the region delineated in white in main text Fig.4a and b. The dashed lines represent the linear regression line, and the blue and red cross sings denote the mean of the scatters for SSP126 and SSP585, respectively.

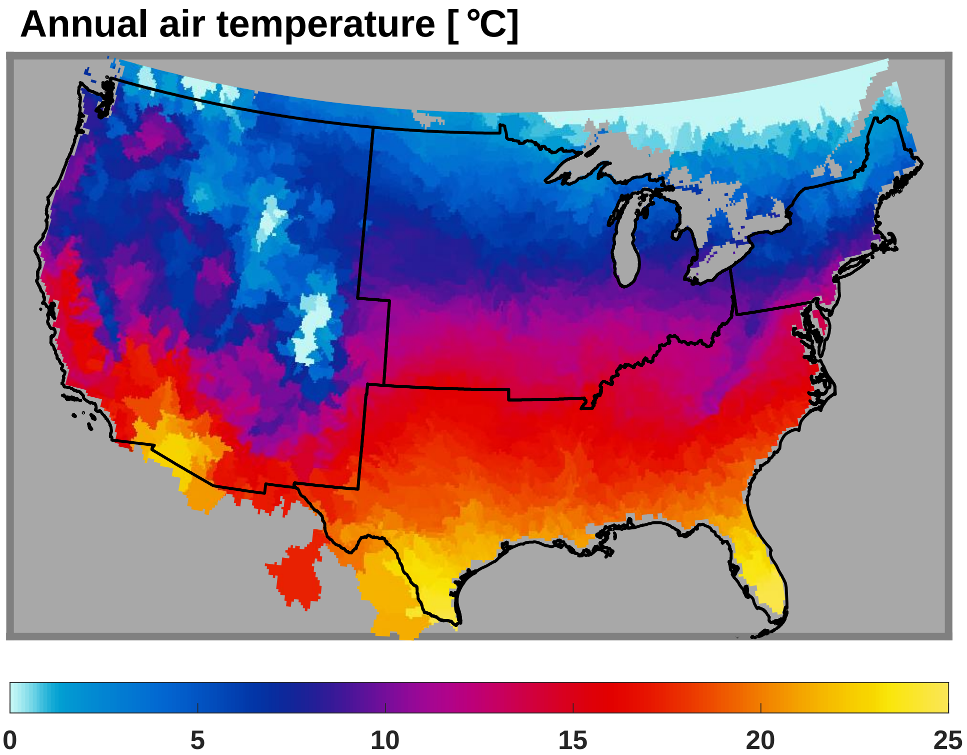

Supplementary Figure 14: Averaged annual air temperature during 1971-2000.

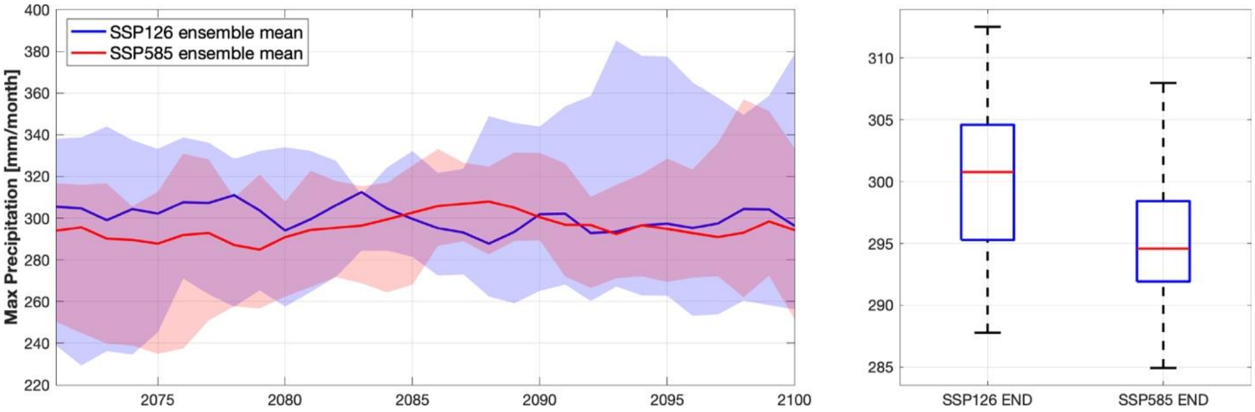

Supplementary Figure 15: Maximum monthly precipitation dynamics over Florida region. Subplot (a) shows the annual time series of maximum monthly precipitation during the end-century period (2071-2100). The shaded areas denote the corresponding 5%-95% of the multi-model ensemble. Subplot (b) shows the corresponding box plots for the multi-model ensemble mean.

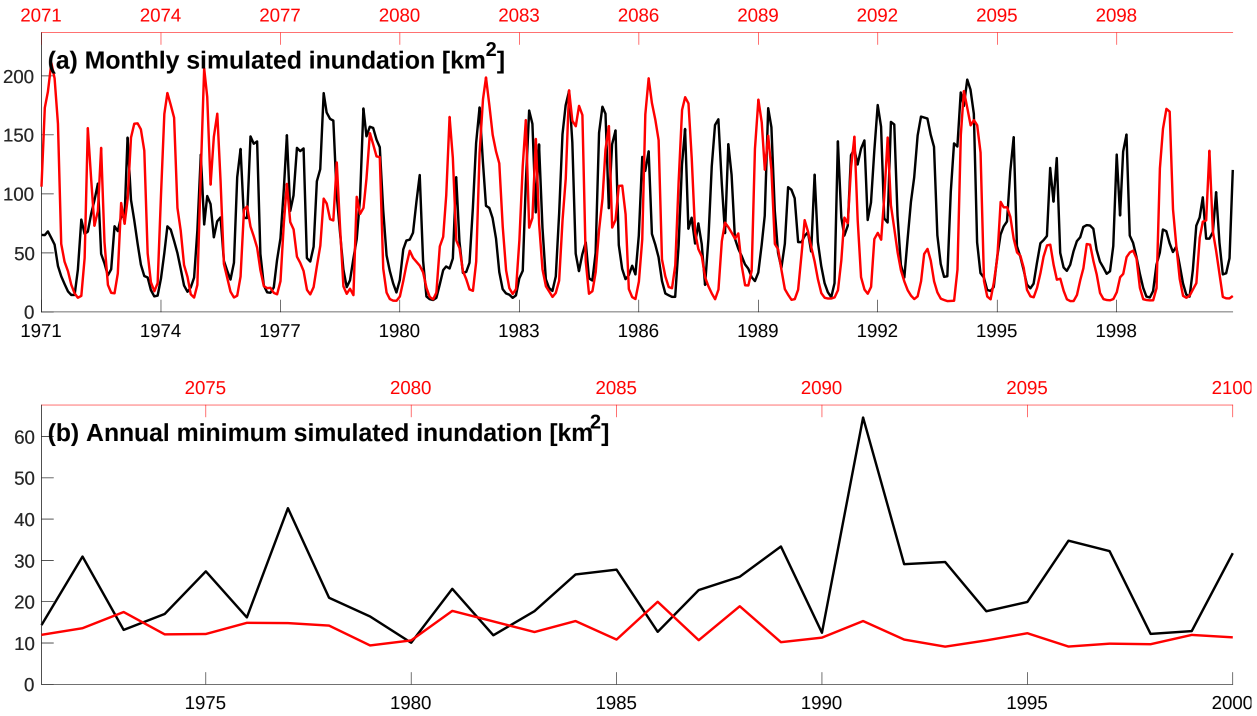

Supplementary Figure 16: The monthly and annual minimum simulated inundation for an example watershed. (a) Simulated total inundation areas of a watershed at monthly scales from historical period (1971-2000 on the bottom X-Axis) and future period under SSP585 scenario (2071-2100 on the top X-Axis). Subplot (b) shows the annual minimum total inundation areas of a watershed scales from historical period (1971-2000 on the bottom X-Axis) and future period under SSP585 scenario (2017-2100 on the top X-Axis). The selected watershed is USGS 08020401 Lower Arkansas watershed and locates at Lon: -91.6587°, Lat: 34.2740°.

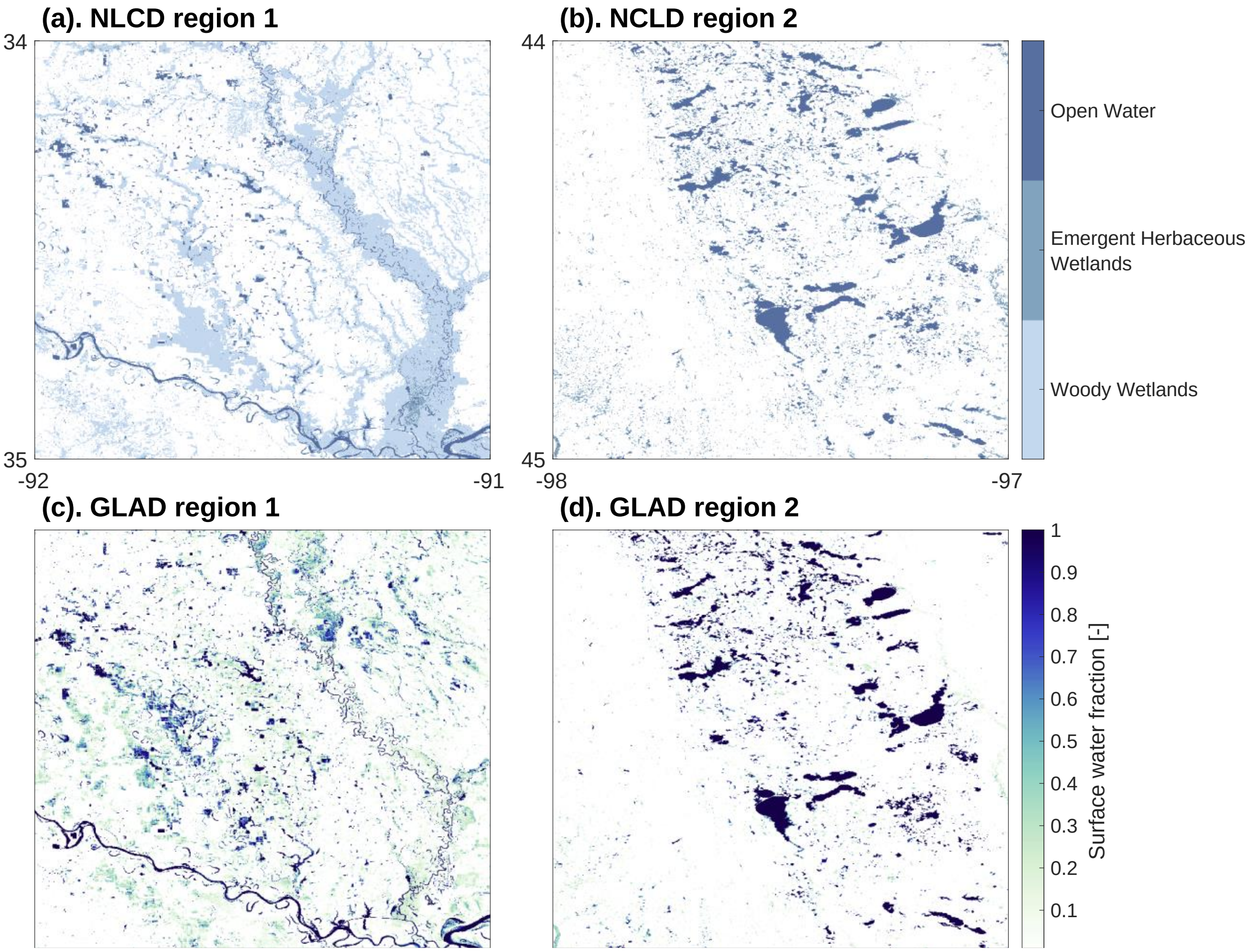

Supplementary Figure 17: **Comparison between National Land Cover Dataset (NLCD) and Global Land Analysis & Discovery (GLAD) surface water dynamics for two $1^\circ \times 1^\circ$ regions.** Subplots (a) and (b) are from NLCD, and subplots (c) and (d) are from GLAD for the corresponding regions.

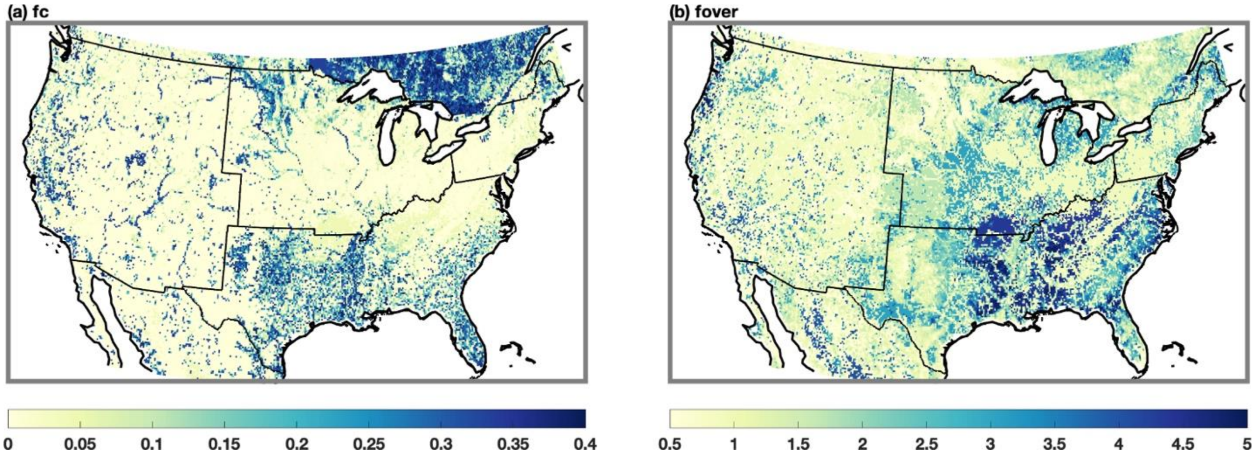

Supplementary Figure 18: The distribution of the calibrated parameter values for (a) f_c and (b) f_{over}.

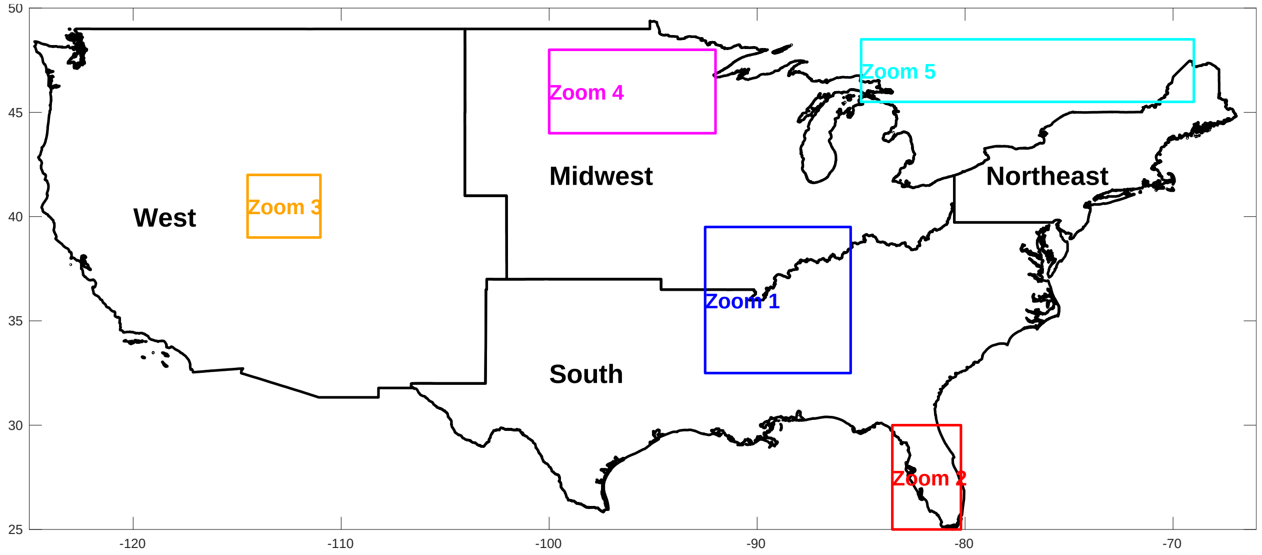

Supplementary Figure 19: **Subregions of continental United States and zoomed-in regions used in Supplementary Figure 5.**

Supplementary References

1. Oleson K, *et al.* Technical description of version 4.5 of the Community Land Model (CLM).) (2013).

2. Zhang Z, *et al.* Cooling effects revealed by modeling of wetlands and land-atmosphere interactions. *Water Resour Res*, e2021WR030573 (2022).
